# Religious Affiliation and Reported Dual Menstrual Material Use Among Young Women in India

**DOI:** 10.3390/healthcare14182893

**Published:** 2026-09-08

**Authors:** Seetharaman Narayanan, Rajeshwari A. Biradar, Yogeswaran Alias Nehru Sivasubramanian, Shyamkumar Sriram, Shiva S. Halli, Jang Bahadur Prasad

**Affiliations:** 1 School of Public Health, Sri Balaji Vidyapeeth (Deemed to be University), Puducherry 607402, India; seethahere@gmail.com (S.N.);; 2 Rehabilitation and Health Services, College of Public Affairs and Health Sciences, University of North Texas, 410 Ave. C, Suite 289, Denton, TX 76203, USA; 3 College of Community and Global Health, University of Manitoba, Winnipeg, MB R3E 0T6, Canada; 4 Department of Biostatistics, Jawaharlal Institute of Postgraduate Medical Education and Research (JIPMER), Puducherry 605006, India

**Keywords:** menstrual hygiene management (MHM), menstrual hygiene products, health behaviour, religion, Muslim women, NFHS-V, India

## Abstract

**Background:** Dual-method menstrual hygiene use, defined as the use of both modern menstrual hygiene products (e.g., sanitary pads) and traditional materials (e.g., cloth), remains under-researched among young women in India. While socioeconomic factors are associated with menstrual practices, religious affiliation may also be associated with differences in reported material use. This study examined the association between religious affiliation and dual-method use among women aged 15–24 years using nationally representative survey data supplemented by contextual evidence from the qualitative and contextual literature. **Methods:** Quantitative analysis used nationally representative National Family Health Survey-V (NFHS-V, 2019–21) data for 240,230 women aged 15–24 years. Survey-weighted bivariate and multivariable logistic regression analyses estimated the prevalence and adjusted odds of reported dual-method use across sociodemographic and religious groups. Seven purposively selected qualitative and contextual sources were used to provide contextual insights into the quantitative findings. **Results:** Muslim women had the highest prevalence of reported dual-method use (31.7%), compared with 27.5% among Hindu women and 21.6% among other religious groups. The association between religious affiliation and dual-method use was also observed across several demographic subgroups. Among women with improved toilet facilities, dual-method use was higher among Muslim women than Hindu women (32.3% vs. 27.0%). Similarly, among women with higher education, prevalence was 30.1% among Muslim women compared with 23.3% among Hindu women. After adjustment for measured sociodemographic, socioeconomic, structural, and regional characteristics, Muslim women had 36% higher odds of reported dual-method use than Hindu women (AOR 1.36, 95% CI: 1.29–1.43; *p* < 0.001). The qualitative and contextual literature provided contextual insights into the potential roles of privacy, household expectations, and disposal concerns in menstrual material use. However, NFHS-V cannot establish whether materials were used concurrently or sequentially or identify the specific contexts of use. **Conclusions:** Religious affiliation was associated with dual-method menstrual hygiene use after adjustment for socioeconomic and demographic characteristics. The findings indicate that menstrual health interventions should address socioeconomic barriers alongside the social and cultural contexts in which menstrual practices occur. Community-informed approaches incorporating privacy, disposal, household norms, and infrastructure may complement product provision and contribute to more equitable menstrual health outcomes.

## 1. Introduction

Menstrual Hygiene Management (MHM), increasingly recognized through the broader lens of Menstrual Health and Hygiene (MHH), is a fundamental pillar of women’s reproductive health, bodily autonomy, and psychosocial well-being [1,2]. In India, despite significant policy momentum, MHM remains a persistent and multi-layered public health challenge, particularly among the 113 million adolescent girls who navigate deep-rooted cultural taboos alongside structural deficits [3,4,5]. While national trends show a gradual increase in the adoption of hygienic products, use varies across socioeconomic, educational, and cultural contexts [3,5,6]. Menstrual hygiene practices are also relevant to health and well-being, with studies in regions like Odisha demonstrating a strong association between cloth or no material practices and a higher prevalence of lower reproductive tract infections (RTIs), such as bacterial vaginosis and candidiasis [6].

Current global monitoring frameworks, such as the WHO/UNICEF Joint Monitoring Programme (JMP), define adequate MHM through the use of clean materials, private changing spaces, and soap and water for bathing [1,2,7]. However, evidence from rural and urban India suggests that these criteria fail to capture the lived experience of menstruation, which many women describe as a “burden on the head”—a complex negotiation of physical discomfort, social support needs, and a supportive sociocultural environment [7]. Infrastructure alone, such as the presence of a household toilet, does not guarantee exclusive hygienic practice; approximately 20% of women with access to toilets refrain from using them during their periods due to fear of staining or lack of disposal facilities [7,8]. This indicates that “one-size-fits-all” interventions focusing solely on product distribution are insufficient to address the deep-seated psychosocial and environmental barriers [4,9,10].

A significant barrier to effective MHM is the culture of silence and the pervasive “menstrual etiquette” that requires girls to keep their menstrual status hidden from boys and men [4,10]. This stigma often results in limited pre-menarche awareness; in India, only about half of adolescent girls are informed about menstruation before its onset [5,11]. Mothers and sisters remain the primary, yet often ill-equipped, sources of information, while the role of teachers is frequently reported as negligible or hampered by their own discomfort with the subject [1,4,12]. Consequently, many girls enter puberty with fear and confusion, often perceiving menstruation as a disease or divine punishment rather than a natural biological process [4,10,13].

An important but relatively underexplored aspect of menstrual material use is the reported use of materials from more than one material category. In the present study, dual menstrual material use refers to respondents reporting materials from both predefined categories. Because NFHS-V does not record the timing, frequency, sequence, or setting of material use, this measure does not establish whether materials were used concurrently or sequentially [3,14]. Religious affiliation may also be associated with differences in menstrual material use. Existing evidence from India suggests that menstrual practices vary across social and cultural groups, but nationally representative evidence examining the association between religious affiliation and reported dual menstrual material use among young women remains limited [3,11].

The Social Ecological Model and the Theory of Planned Behavior provide useful frameworks for considering how menstrual health practices may be influenced by individual, interpersonal, community, and structural factors. In the Indian context, these factors may influence how menstruation is understood and managed at the household level, including preferences and constraints related to menstrual materials. There is limited nationally representative evidence on whether differences in reported menstrual material use by religious affiliation persist after accounting for measured socioeconomic and demographic characteristics.

The recent literature has documented the role of social identities such as caste and religion in shaping menstrual hygiene practices in India [15,16]. These studies suggest that religious affiliation operates as a social marker reflecting household norms, gendered expectations, privacy practices, and disposal constraints, rather than as a direct causal determinant of product choice. However, large-scale quantitative evidence on how these identity-linked norms intersect with the use of multiple menstrual materials remains limited. Accordingly, this study focuses on religious affiliation not to essentialize communities, but to better understand how socially embedded norms and lived practices may influence menstrual hygiene behaviour across diverse populations. By examining these patterns at a national level, the study contributes evidence toward identifying culturally specific barriers and informing more context-sensitive menstrual health interventions and policies.

Therefore, this study aimed to examine the association between religious affiliation and reported dual menstrual material use among young women aged 15–24 years in India using nationally representative NFHS-V data. Specifically, we examined differences in reported dual menstrual material use across religious groups and assessed whether the association between religious affiliation and the outcome persisted after adjustment for measured sociodemographic and socioeconomic characteristics. The selected qualitative and contextual literature was used to contextualise the observed quantitative associations and identify possible explanations that could be examined in future research.

## 2. Methods

### 2.1. Study Design: Quantitative Analysis with Qualitative Contextualisation

**Figure 1:**
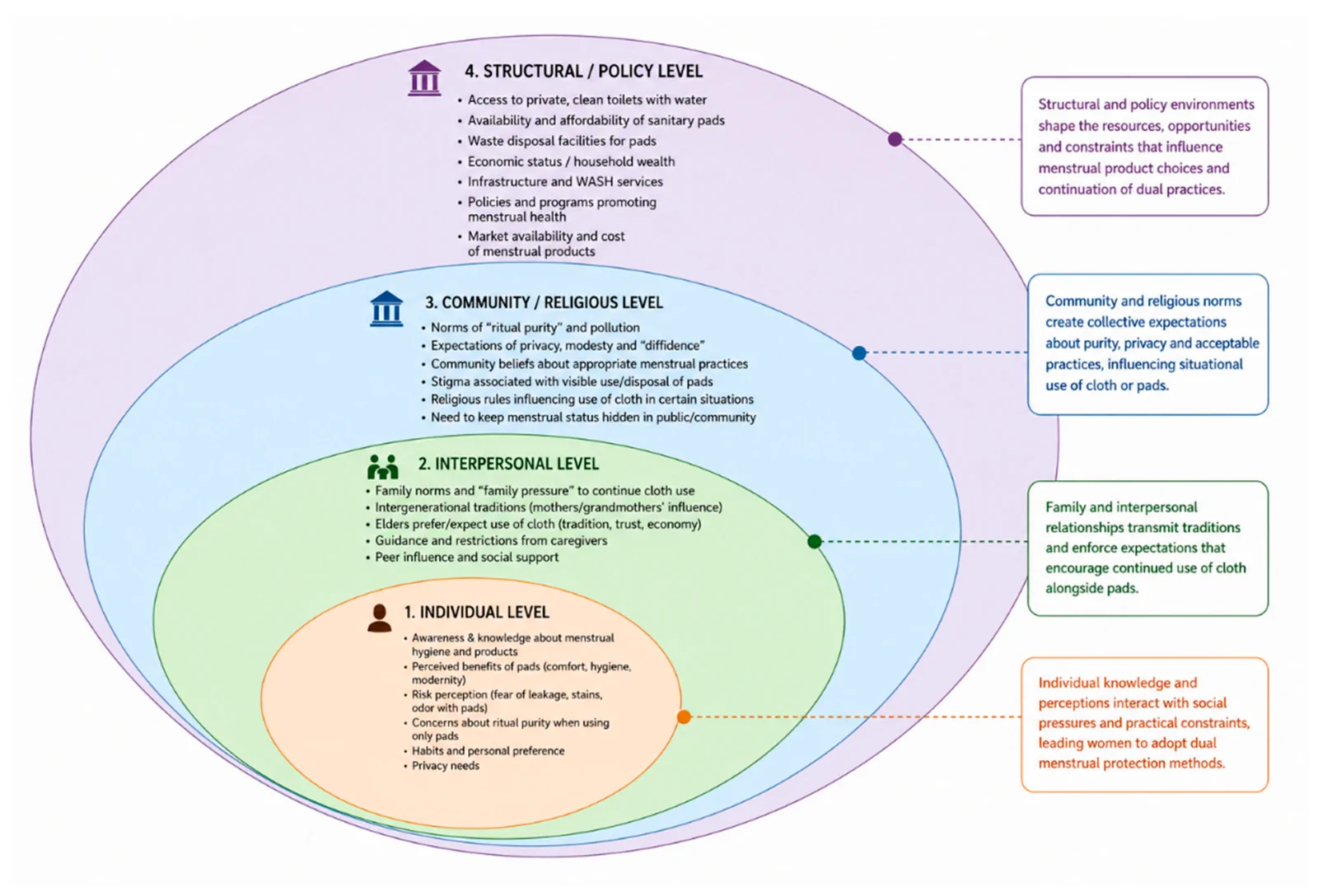
Social Ecological Model (SEM) of determinants influencing dual methods.

This study used a cross-sectional design based on data from the fifth round of the National Family Health Survey (NFHS-V), conducted during 2019–2021. NFHS-V is a nationally representative household survey conducted using a multistage, stratified sampling design. The present analysis used data for women aged 15–24 years from the individual women’s dataset. The analysis accounted for sampling weights, clustering, and stratification using the survey (svy) commands in Stata/SE 19.0 (StataCorp LLC, College Station, TX, USA). The study adopts a quantitative analytical framework based on nationally representative survey data, complemented by an interpretive synthesis of the purposively selected qualitative and contextual literature. This approach is informed by the Social Ecological Model (SEM) and the Theory of Planned Behavior (TPB), which conceptualize health behaviours as being shaped not only by individual choice but also by structural resources and normative cultural contexts. The qualitative and contextual literature is used to contextualise the quantitative findings by linking the observed patterns (the “what”) with plausible behavioural and cultural explanations (the “why”), rather than to provide confirmatory evidence. This study was conceptually informed by the SEM, which considers menstrual hygiene practices as being influenced by interacting individual, interpersonal, community, and structural factors (Figure 1).

### 2.2. Quantitative Data Source: NFHS-V (2019–2021)

The quantitative data were extracted from the fifth round of the NFHS-V, a massive, multi-stage stratified survey conducted across 707 districts in all 28 states and 8 Union Territories of India, using a two-stage sampling design based on the 2011 Census. In the first stage, Primary Sampling Units (PSUs)—villages in rural areas and Census Enumeration Blocks in urban areas—were selected using probability proportional to size (PPS) sampling. In the second stage, 22 households were systematically selected from each PSU. From a total survey pool of 724,115 women, the current analysis focuses on a specific sub-sample of 241,181 young women aged 15–24 years. Among 241,181 eligible respondents, 240,230 had complete information on dual-method use and were included in the analysis. The present analysis focused on women aged 15–24 years, as information on menstrual material use in NFHS-V was collected for this age group. NFHS-V used a multistage, stratified sampling design, and the present analysis accounted for the survey sampling design when estimating population-level associations and their variance.

### 2.3. Operationalization of Outcome Variable

The primary outcome was reported dual menstrual material use, defined as the reported use of materials from both predefined menstrual-material categories. The categories comprised modern menstrual hygiene products (sanitary pads, menstrual cups, and tampons) and cloth or no material. Respondents reporting materials from only one category were classified as single-category users, whereas those reporting materials from both categories were classified as dual-material users. This classification reflects the types of materials reportedly used by the respondents and does not establish whether different materials were used concurrently or sequentially. The NFHS-V questionnaire does not provide information on the timing, frequency, duration, sequence, or setting of use. Therefore, dual menstrual material use was not interpreted as directly measured switching behaviour or context-specific substitution. For the present analysis, single-category use comprised respondents who reported materials from only one predefined category: either modern menstrual hygiene products (sanitary pads, menstrual cups, or tampons) or cloth/no material. Reported dual menstrual material use comprised respondents who reported materials from both categories. Accordingly, throughout this study, “reported dual menstrual material use” refers specifically to an analytical classification based on reported material categories and should not be interpreted as evidence of a particular behavioural mechanism.

### 2.4. Explanatory Variables

Explanatory variables were selected to represent sociodemographic, socioeconomic, and structural characteristics that may be associated with reported dual menstrual material use:

- Religion: Categorised into Hindu, Muslim, and Others (including Christians, Sikhs, Buddhists, and other reported religious affiliations), with Hindu women serving as the reference category in the multivariable analysis.
- Socio-Demographic: Age (15–19 and 20–24), place of residence (urban/rural), current marital status (never/ever married), and caste/social group (Scheduled Caste (SC), Scheduled Tribe (ST), Other Backward Classes (OBC), and none of these groups).
- Socio-Economic: Educational attainment (no education, primary, secondary, and higher) and household wealth status assessed using the NFHS-V wealth index and categorised into five quintiles (poorest, poorer, middle, richer, and richest).
- Structural and information-access characteristics: Exposure to mass media (yes/no) and type of toilet facility (improved/unimproved).
- Geographic region: Region of residence was categorized into six groups—North, Central, East, Northeast, West, and South.

### 2.5. Literature-Grounded Interpretive Framework

To contextualise the quantitative findings from the NFHS-V, a non-systematic narrative contextualization of the secondary literature was conducted. The purposively selected sources comprised a mix of primary qualitative studies, systematic qualitative meta-syntheses, and programmatic landscape reports on menstrual hygiene in India and similar low- and middle-income countries (LMICs). These included primary qualitative research from Odisha [7] and urban India [9], a global systematic qualitative meta-synthesis [14], and programmatic or contextual reports from Dasra [4], WaterAid [8], Sommer et al. (2015) [10], and UNICEF (2019) [2].

The analysis of these sources did not involve primary data collection. Instead, a thematic mapping exercise was undertaken. Recurring themes related to ‘privacy constraints,’ ‘disposal anxiety,’ ‘menstrual taboos,’ and ‘material switching’ were extracted from these sources and considered alongside the quantitative findings to provide contextual interpretations of reported dual menstrual material use. The themes were treated as possible explanations informed by the previous literature rather than as mechanisms directly measured or established in the NFHS-V data. The secondary literature therefore provides contextual plausibility rather than formal methodological triangulation.

### 2.6. Statistical Analysis

The statistical analysis was performed using Stata/SE 19.0 using survey-weighted analysis procedures. The sampling weights provided by NFHS-V were used to account for the complex survey design. Survey-weighted analyses incorporated sampling weights (wt), stratification (V023), and primary sampling units (V021), with variance estimates obtained using Taylor series linearization. The weighted prevalence estimates and 95% confidence intervals (CIs) were estimated for the reported usage of dual-use menstrual hygiene products. The descriptive analysis included 240,230 women, with 30,017 PSUs and 2710 strata; the corresponding survey-weighted population size was 241,045.04. The multivariable regression analysis included 218,843 women, with 29,612 PSUs and 2710 strata; the corresponding survey-weighted population size was 218,345.85. The bivariate associations between reported dual-method use and sociodemographic and religious characteristics were evaluated with the survey-weighted tabulation procedure and design-adjusted Rao–Scott tests. The survey-weighted logistic regression procedure was used to obtain adjusted odds ratios (AORs) with 95% confidence intervals (CIs) for factors associated with reported dual-method menstrual hygiene use (yes/no), adjusting for the above factors in a multivariable survey-weighted logistic regression model. The reference category was Hindu religion. For the multivariable analysis, a complete-case approach was used; observations with missing values for any variable included in the respective regression model were excluded from that analysis. The survey-weighted population size reported by Stata represents the sum of the applied survey weights for the analytic sample and should not be interpreted as an actual national population estimate. Multicollinearity was assessed using variance inflation factors (VIFs); the mean VIF was 1.90, with the highest VIF being 4.00, indicating no substantial multicollinearity among the explanatory variables. Model fit was assessed using the survey-adjusted goodness-of-fit test, which indicated evidence of lack of fit (F(9, 26,894) = 2.33; *p* = 0.0128). Design-adjusted tests of formal interaction terms between religious affiliation and selected covariates (residence, education, and household wealth) were conducted, and the predicted probability approach was used to facilitate interpretation of the statistically significant interactions. *p* < 0.05 was deemed statistically significant.

## 3. Results

Among 241,181 eligible women aged 15–24 years, 240,230 had complete information on the primary outcome and were included in descriptive analyses. Complete-case analysis for the multivariable model resulted in an analytic sample of 218,843 women. These patterns reflect reported material combinations and should not be interpreted as evidence of specific switching behaviors or contextual usage patterns. Among the 240,230 women included in the descriptive analysis, 114,111 reported modern menstrual materials only, 56,091 reported traditional materials only, and 70,028 reported materials from both categories. After applying survey weights, the corresponding proportions were 49.99% (95% CI: 49.59–50.39), 22.16% (95% CI: 21.85–22.48), and 27.85% (95% CI: 27.51–28.19), respectively. Thus, the non-dual reference group comprised women reporting modern materials only and those reporting traditional materials only. Within the non-dual reference group, modern-product-only users comprised 67.04% of the unweighted sample and 69.28% (95% CI: 68.86–69.70) after applying survey weights, while traditional-material-only users comprised 32.96% and 30.72% (95% CI: 30.30–31.14), respectively.

### 3.1. Religious Differences in Reported Dual Menstrual Material Use

Reported dual menstrual material use was highest among Muslim women (31.7%), compared with Hindu women (27.5%) and women in other religious groups (21.6%); the differences were statistically significant in the bivariate analysis (*p* < 0.001) (Table 1).

Reported dual menstrual material use declined across increasing socioeconomic status among Hindu women, whereas a similar gradient was less pronounced among Muslim women. These descriptive patterns indicate that differences in reported material use were not fully aligned with socioeconomic status alone. However, because the NFHS-V data do not directly measure household norms, privacy expectations, disposal concerns, or religious practices, these factors cannot be established as explanations for the observed differences.

Higher reported dual menstrual material use among Muslim women was observed across most of the sociodemographic subgroups examined (Table 2).

- Among urban women, reported dual menstrual material use was 28.7% among Muslim women compared with 20.0% among Hindu women. Among never-married and ever-married women, the corresponding prevalence among Muslim women was 32.2% and 30.9%, respectively. Reported dual menstrual material use was higher among Muslim women across the educational categories examined, including among women with higher education (30.1% vs. 23.3% among Hindu women).
- Among the wealth categories, women in the “Others” religious group had the highest reported dual menstrual material use in the poorest quintile, whereas Muslim women had the highest prevalence in the remaining wealth quintiles, ranging from 34.9% in the poorer quintile to 28.4% in the richest quintile.
- Among women with improved toilet facilities, reported dual menstrual material use was 32.3% among Muslim women compared with 27.0% among Hindu women.

### 3.2. Multivariable Logistic Regression

Association between religious affiliation and reported dual menstrual material use. To assess the association between religious affiliation and reported dual menstrual material use after adjustment for other measured characteristics, a multivariable logistic regression model was performed. After adjustment for age, education, wealth, media exposure, and other prespecified covariates, religious affiliation remained significantly associated with reported dual menstrual material use. Compared with Hindu women, Muslim women had higher odds of reporting dual-method use (AOR 1.36, 95% CI 1.29–1.43; *p* < 0.001), whereas women belonging to other religious groups had lower odds (AOR 0.91, 95% CI 0.85–0.98; *p* = 0.015) (Table 3). Women in the “Others” religious category had slightly lower odds of reported dual menstrual material use than Hindu women (AOR = 0.91, 95% CI: 0.85–0.98, *p* = 0.015). Other characteristics significantly associated with reported dual menstrual material use included residence and mass-media exposure. Rural women had higher odds than urban women (AOR = 1.29, 95% CI: 1.23–1.36, *p* < 0.001), while women exposed to mass media also had higher odds of reported dual menstrual material use (AOR = 1.29, 95% CI: 1.24–1.34, *p* < 0.001) (Table 3). There was evidence of interaction between religion and residence, education, and wealth regarding the use of dual menstrual materials (*p* for interaction < 0.001, 0.031, and <0.001 respectively). The predicted probabilities of reported dual method use were higher for Muslim women in most groups of wealth, education, and residence compared to Hindu and other women (Table 4). The greatest differences occurred by wealth groups, with the size of the religious difference differing by socioeconomic groups. Compared with the North, higher odds of reported dual menstrual material use were observed in the Central (AOR = 1.78, 95% CI: 1.69–1.87), while lower odds were observed in the South (AOR = 0.74, 95% CI: 0.69–0.79).

### 3.3. Qualitative Contextualisation of the Quantitative Findings

The selected sources describe several social and cultural factors that may provide context for differences in menstrual material use, including privacy, stigma, household expectations, affordability, and disposal concerns. The selected qualitative and contextual sources (Table 5) describe menstrual material use as influenced by context-specific social, cultural, and practical considerations. Key behavioural drivers identified in the sources include:

- Contextual and practical considerations: Women often use sanitary pads in public spaces (schools, markets) for mobility and confidence but revert to cloth at home due to perceived comfort or privacy constraints [7].
- Privacy and menstrual discretion: Differences in reported menstrual-material use may partly reflect sociocultural factors related to menstrual privacy, acceptability, and household practices [7]. However, these factors were not directly measured in the NFHS-V dataset and therefore should be considered possible explanations rather than established mechanisms. The “fear of improper disposal” or the risk of male family members discovering used materials often leads to the situational use of cloth, which can be easily hidden or managed within the household [7,9].
- Psychosocial and household considerations: Qualitative evidence highlights that menstruation is often viewed as a psychosocial stressor. Dual method use allows women to balance the social acceptability of modern products with traditional intergenerational practices that satisfy household scrutiny [7,14].

**Table 5:** Purposively selected qualitative and contextual sources used to contextualise reported dual menstrual material use in the NFHS-V analysis.

Sl. No.	Study (Year)	Location & Method	Key Behavioural/Cultural Factors Identified	Relevance to NFHS Dual Method Findings
1	Hennegan et al., 2019 [14]	Global qualitative meta-synthesis	Pads used outside; cloth preferred at home; disposal concerns; gradual adoption of modern products	The external literature describes dual-method use as a possible transitional pattern; this was not directly assessed in NFHS-V.
2	MacRae et al., 2019 [7]	Odisha, India (in-depth interviews)	Cloth preferred for comfort at home; pads for mobility; fear of improper disposal	The literature describes context-specific material use, including differences between home and public settings; such switching was not directly assessed in NFHS-V
3	Rajagopal & Mathur, 2017 [9]	Urban India (Qualitative study)	Norms of menstrual discretion discussed in religion-specific contexts, hiding pads from men, family restrictions	Provides contextual information on privacy and menstrual discretion
4	House et al., 2013 (WaterAid Report) [8]	Multi-country review	Cultural acceptance of cloth; disposal challenges for pads	Supports that access does not automatically lead to exclusive pad use
5	Dasra Report, 2014 [4]	India (FGDs + surveys)	Girls ration pad use; cloth use at home; economic considerations	Aligns with dual method use driven by cost and household norms
6	Sommer et al., 2015 [10]	Multi-country qualitative	Menstrual secrecy; method switching depending on context	Describes contextual factors associated with menstrual material choices
7	UNICEF, 2019 [2]	Programmatic/qualitative synthesis	Limited private drying space; selective pad use; community norms	Disposal/privacy constraints lead to mixed material use

## 4. Discussion

The findings of this study provide a national perspective on reported menstrual material use among young women in India and highlight an association between religious affiliation and reported dual menstrual material use after adjustment for measured sociodemographic and socioeconomic characteristics. Using nationally representative NFHS-V data from 2019 to 2021, this study examined reported dual menstrual material use among 241,181 women aged 15–24 years. The selected qualitative and contextual literature was used to provide possible interpretations of the observed quantitative associations. The persistence of the association after adjustment indicates that differences in reported dual menstrual material use were not fully accounted for by the measured sociodemographic, socioeconomic, and structural characteristics included in the model. Previous qualitative research has identified factors such as privacy, stigma, household expectations, affordability, and disposal concerns as potentially relevant to menstrual material choices; however, these factors were not directly measured in the NFHS-V analysis. In this study, the qualitative and contextual literature is used solely to contextualize observed associations and generate plausible interpretive explanations, rather than to provide confirmatory evidence or substitute for primary qualitative research.

### 4.1. Religious Affiliation as an Associated Social Marker of Dual Menstrual Material Use

A central finding is that in this nationally representative sample, reported dual menstrual material use was higher among Muslim women (31.7%) than among Hindu women (27.5%) and women in the Others category (21.6%). After adjustment for the measured sociodemographic, socioeconomic, structural, and regional characteristics included in the model, Muslim women had higher odds of reported dual-method use than Hindu women (AOR = 1.36, 95% CI: 1.29–1.43). This persistent association indicates that differences were not fully accounted for by the measured sociodemographic and socioeconomic characteristics. It may also reflect differences in cultural norms, beliefs, and social expectations; however, these factors were not directly measured in the present study [14,17].

The analysis further reveals important variations across wealth quintiles and place of residence. Among Muslim women, dual-method use remained relatively high across wealth quintiles, ranging from 28.4% in the richest quintile to 34.9% in the poorer quintile, without a consistent linear gradient. In contrast, Hindu women demonstrate a much steeper decline, with prevalence reducing to 16.6% among the richest group. Similarly, a distinct rural-urban differential is observed. Hindu women show a substantial gap between rural and urban residence (30.3% vs. 20.0%), whereas the difference among Muslim women is comparatively smaller, at approximately 4.9 percentage points. In the caste/social-group stratification, reported dual-method use was 34.3% among Muslim women classified as OBC. These findings further indicate that religious and socioeconomic characteristics may intersect in relation to reported menstrual material use/practices.

These findings suggest that economic advancement and urban residence alone may not necessarily translate into exclusive adoption of hygienic menstrual products. The persistence of religious differentials in menstrual hygiene practices, even after accounting for wealth quintiles and rural–urban residence, indicates that health behaviours are also potentially shaped by other social and cultural factors not directly measured in the survey. Interaction analyses indicated that the association between religious affiliation and dual-method use varied significantly by residence, education, and wealth (*p*-interaction < 0.001, 0.031, and <0.001, respectively). The corresponding predicted probabilities are presented in Table 4. Religious affiliation may therefore capture aspects of shared cultural norms, beliefs, and expectations. Such culturally embedded norms could potentially contribute to the continued concurrent use of traditional cloth alongside modern sanitary pads, although the present study cannot establish these mechanisms.

### 4.2. Dual Method Use as a Potential Transitional Pattern

The selected literature suggests that dual-method use may represent an intermediate or transitional pattern in some contexts; however, this interpretation was not directly assessed in the NFHS-V data. While the adoption of modern menstrual hygiene products (like sanitary pads) is increasing, traditional materials such as cloth may continue to be used alongside modern products [5]. As highlighted by the qualitative studies in Table 5, this pattern may be consistent with situational or context-dependent use of different menstrual materials, as suggested by the previous qualitative and contextual literature; however, such mechanisms were not directly measured in NFHS-V [10,14]. Previous qualitative studies have reported that menstrual product choices may vary across settings and may be influenced by factors such as mobility, comfort, cost, and family or social expectations [1,7,14]. Reported dual-method use may be compatible with context-dependent use described in the qualitative and contextual literature, but NFHS-V cannot establish such behaviour [14].

### 4.3. The Possible Sociocultural Explanations and “Menstrual Etiquette”

Modesty norms, privacy expectations, disposal concerns, and household decision-making were not directly measured in NFHS-V. Religious affiliation is therefore considered here as a social marker associated with unmeasured contextual factors, rather than as a direct measure of these factors. The discussion below draws on the external qualitative and contextual literature, which does not exclusively represent Muslim populations and cannot be linked directly to NFHS-V respondents; these studies are used only to provide interpretive context and generate plausible explanations. With this caveat, the higher prevalence of reported dual-method use among Muslim women may be consistent with sociocultural considerations related to bodily privacy, modesty, and household discretion described in the external qualitative and contextual literature; however, these factors were not measured in NFHS-V and cannot be considered explanations demonstrated by the present study [3,14].

Previous qualitative studies from India describe menstruation as being embedded within a “culture of silence,” in which women may be expected to maintain secrecy regarding menstrual processes, particularly in the presence of male family members [9,10]. Within such contexts, concerns regarding the visible disposal of sanitary pads may influence menstrual management practices, including the continued use of cloth, which may be perceived as easier to conceal or manage privately within the household [7,14]. The ethnographic literature further characterizes the management of menstrual visibility as a potential psychosocial burden, with perceptions of social acceptability influencing menstrual management practices [4,7]. These findings provide possible contextual interpretations for the observed differences in reported menstrual material use across religious groups; however, the underlying mechanisms were not directly measured in the present study and therefore remain hypotheses requiring further investigation.

### 4.4. Education and Infrastructure in Relation to Reported Dual Material Use

One of the most striking results is that formal education does not show a consistent inverse relationship with dual method use across all religious groups. Muslim women reported the highest dual-use rates across every educational level, including those with “Higher” education (30.1%) compared to Hindu women (23.3%). This pattern may indicate that education alone does not fully account for differences in menstrual hygiene practices across religious groups. Previous qualitative research has identified familial and community norms, beliefs surrounding menstruation, privacy concerns, and intergenerational transmission of practices as potential influences on menstrual hygiene behaviour [3,9]. Similarly, access to improved sanitation facilities was not consistently associated with exclusive use of modern menstrual hygiene products [8,14]. Muslim women had the highest rate of dual use (32.3%) even when they had access to improved toilets. This can be considered in relation to the Theory of Planned Behavior, which emphasizes the role of perceived behavioural control in shaping health behaviours [18]. However, the present study did not directly measure perceived behavioural control, privacy, or disposal-related concerns, and therefore these mechanisms should be interpreted cautiously.

### 4.5. Theoretical Implications: The Social Ecological Model

The persistence of dual menstrual hygiene practices can be understood through the SEM, which emphasizes that health behaviours are shaped by interacting influences across multiple levels [19].

At the Individual Level, increasing knowledge and awareness regarding menstrual hygiene may support the adoption of sanitary pads and other modern menstrual hygiene products [5]. Women recognize the benefits of pads in terms of comfort, hygiene, and convenience; however, individual concerns related to privacy, fear of leakage, and stigma continue to influence menstrual product choices [14].

At the Interpersonal Level, family norms, intergenerational traditions, and social expectations may shape menstrual practices. The continued use of cloth is often reinforced [4,12,17]. These interpersonal influences may contribute to the continuation of dual menstrual hygiene practices despite increasing awareness of hygienic methods.

At the Community/Religious Level, the external literature describes normative expectations surrounding ritual purity, modesty, and menstrual discretion that may influence menstrual material practices [9,10]. However, these factors were not directly measured in NFHS-V and should therefore be considered possible contextual explanations rather than demonstrated pathways.

Moreover, an important observation from this study was the observed religious differences despite similarities in socioeconomic status and educational attainment. The higher prevalence of dual-method use among Muslim women may reflect differences in social and cultural contexts; however, the present dataset did not directly measure community expectations, concealment practices, disposal concerns, or religiously specific menstrual restrictions. Therefore, these mechanisms should be considered as possible explanations rather than demonstrated pathways.

At the Structural/Policy Level, broader contextual factors such as access to private toilets, availability of water and disposal facilities, affordability of sanitary products, and household economic status may influence menstrual practices [5]. Inadequate sanitation infrastructure, lack of privacy, and financial limitations often restrict the exclusive use of hygienic menstrual products, particularly in low-resource settings [10]. Consequently, women may continue using cloth alongside sanitary pads as a practical and economically viable strategy, thereby sustaining dual menstrual protection practices [19].

The conceptual framework presents multi-level factors potentially associated with the persistence of dual-method menstrual hygiene practices, defined as the use of modern menstrual hygiene products alongside traditional materials such as cloth or, where applicable, no material, conceptualized using the SEM. The concentric layers represent interacting individual, interpersonal, community/religious, and structural factors that may influence menstrual hygiene practices.

### 4.6. Media Exposure and Information Access

Mass-media exposure was positively associated with reported dual-method use in the adjusted analysis (AOR 1.29, 95% CI: 1.24–1.34). This association should not be interpreted as evidence that media exposure promotes dual-method use. The NFHS-V variable does not capture the type, frequency, content, or menstrual-health relevance of media exposure. One possible interpretation is that exposure to information may coexist with continued use of multiple menstrual materials, but this mechanism cannot be assessed using the available data. The finding highlights the need for future research to examine not only exposure to information but also its content, relevance, and relationship to household and practical constraints [10]. However, because the present study did not assess the content or type of media exposure, these mechanisms cannot be directly established from the data.

### 4.7. Disposal Concerns and Infrastructure Limitations

Concerns about the disposal of used menstrual materials may influence menstrual product choices, particularly where privacy and appropriate disposal facilities are limited. The inadequacy of school sanitation infrastructure, specifically the absence of sealed waste bins, private changing rooms, and incinerators, has been identified in previous research as a potential barrier to the exclusive use of modern menstrual hygiene products. Furthermore, limited discussion of menstrual management and disposal within families and schools may further constrain girls’ ability to use and dispose of menstrual products comfortably and privately. However, these specific mechanisms were not directly measured in the present study and should therefore be considered as potential explanations rather than established determinants of dual-method use. These factors may be relevant to menstrual material choices in some settings, but their contribution to the observed religious differences could not be assessed in the present study.

### 4.8. Limitations

This study is based on cross-sectional NFHS-V data and therefore identifies statistical associations rather than causal relationships. The analysis does not include direct measures of modesty, religious norms, household negotiations, or decision-making processes related to menstrual management; consequently, religious affiliation may reflect a constellation of unmeasured household-level and normative factors such as gender norms, privacy constraints, disposal practices, and intra-household regulation of menstruation. In addition, menstrual hygiene practices are self-reported and may be subject to recall bias or social desirability bias. While the qualitative and contextual literature was used to contextualize the findings, this synthesis is based on secondary studies rather than primary fieldwork and does not exclusively represent Muslim communities, nor can the reported qualitative experiences be directly attributed to the NFHS-V respondents. The binary outcome also combined modern-product-only users and cloth/no-material-only users within the non-dual reference group; these groups represent substantially different menstrual material practices, and their heterogeneity may limit the interpretation of comparisons between dual and non-dual users. The goodness-of-fit test indicated evidence of some model misfit (F(9, 26,894) = 2.33, *p* = 0.0128); therefore, the adjusted associations should be interpreted cautiously, as potential nonlinearities, interactions, or other unmeasured factors may not have been fully captured.

### 4.9. Conclusions

The findings suggest that “one-size-fits-all” menstrual health interventions may be insufficient in India’s diverse social context [8]. Religious affiliation remained associated with reported dual-method use after adjustment for socioeconomic and demographic characteristics, with Muslim women having higher odds of reported dual-method use than Hindu women (AOR 1.36, 95% CI 1.29–1.43). However, the cross-sectional design does not establish causality, and the underlying sociocultural mechanisms were not directly measured in NFHS-V.

Reported dual-method use varied across wealth categories, with a non-linear pattern that differed by religious group, suggesting that menstrual health interventions may benefit from attention to socioeconomic as well as diverse social and cultural contexts. Moving beyond product provision toward community-informed approaches that consider privacy, disposal practices, household circumstances, and menstrual-health infrastructure may help support more equitable menstrual health outcomes. However, these contextual factors were not directly measured in the present analysis and should therefore be considered potential areas for further investigation rather than demonstrated explanations of the observed associations.

## Figures and Tables

**Table 1:** Reported dual menstrual material use by religious affiliation among women aged 15–24 years in India.

Characteristic	Unweighted n	Dual-Method Use, Weighted % (95% CI)	χ^2^/F (*p*-Value)
Hindu	180,745	27.5 (27.11–27.84)	492.32/82.36 (<0.001)
Muslim	33,662	31.7 (30.73–32.66)	
Others	25,823	21.6 (20.44–22.72)	
Total	240,230	27.9 (27.51–28.19)	

Note: Percentages are survey-weighted estimates with 95% confidence intervals. χ^2^ represents the uncorrected Pearson chi-square statistic, and F represents the design-adjusted F statistic.

**Table 2:** Bivariate distribution of dual-method use among 240,230 Indian women aged 15–24 years by religion and selected sociodemographic characteristics.

Variable and Category	Hindu			Muslim			Others		
	Total	% (95% CI)	χ^2^/F (*p*-Value)	Total	% (95% CI)	χ^2^/F (*p*-Value)	Total	% (95% CI)	χ^2^/F (*p*-Value)
Age in 5-year groups									
15–19	91,141	27.6 (27.1–28.0)	0.790/0.478 (0.489)	17,466	31.7 (30.6–32.8)	0.001/0.000 (0.985)	13,073	21.0 (19.7–22.4)	3.964/1.317 (0.251)
20–24	89,604	27.4 (26.9–27.8)		16,196	31.7 (30.5–32.9)		12,750	22.1 (20.7–23.5)	
Type of place of residence									
Urban	38,264	20.0 (19.3–20.7)	1917.649/499.746 (<0.001)	10,539	28.7 (27.1–30.3)	89.838/24.049 (<0.001)	5603	13.2 (11.7–14.9)	532.729/109.010 (<0.001)
Rural	142,481	30.3 (29.9–30.7)		23,123	33.6 (32.6–34.7)		20,220	25.7 (24.5–27.1)	
Current marital status									
Never Married	115,779	27.5 (27.1–28.0)	0.767/0.416 (0.519)	22,482	32.2 (31.1–33.3)	6.541/2.830 (0.093)	19,133	20.8 (19.7–22.0)	21.015/6.252 (0.012)
Ever Married	64,966	27.4 (26.9–27.9)		11,180	30.9 (29.5–32.2)		6690	23.4 (21.6–25.4)	
Educational level									
No education	11,497	24.4 (23.3–25.5)	497.005/90.488 (<0.001)	3394	31.1 (28.9–33.3)	7.366/1.262 (0.285)	1060	22.8 (19.2–26.9)	173.151/20.700 (<0.001)
Primary	10,449	28.2 (27.1–29.4)		2930	31.2 (28.9–33.5)		1628	26.6 (22.9–30.7)	
Secondary	125,041	28.9 (28.5–29.3)		23,163	32.1 (31.1–33.2)		19,153	23.2 (21.9–24.5)	
Higher	33,758	23.3 (22.7–24.0)		4175	30.1 (28.0–32.3)		3982	15.6 (14.0–17.5)	
Type of social groups									
Scheduled caste/Scheduled tribe	69,717	28.4 (27.9–29.0)	373.091/71.901 (<0.001)	2691	28.9 (25.9–32.1)	65.788/13.623 (<0.001)	20,738	23.0 (21.8–24.3)	28.740/3.103 (0.046)
OBC	77,817	28.7 (28.2–29.2)		14,001	34.3 (33.0–35.5)		1782	20.4 (17.6–23.5)	
None of them	28,526	23.1 (22.3–23.9)		9847	29.7 (28.0–31.4)		2524	19.9 (17.8–22.2)	
Household wealth index									
Poorest	40,533	29.3 (28.6–29.9)	2812.204/312.084 (<0.001)	6381	29.2 (27.5–31.0)	87.223/8.717 (<0.001)	5717	32.0 (29.6–34.5)	760.254/53.212 (<0.001)
Poorer	42,978	33.7 (33.0–34.4)		7666	34.9 (33.2–36.7)		6409	29.4 (27.0–31.8)	
Middle	38,826	29.9 (29.2–30.6)		6944	32.8 (31.0–34.7)		5311	25.2 (22.8–27.8)	
Richer	32,908	24.9 (24.1–25.6)		7431	32.2 (30.5–34.0)		4293	18.6 (16.5–20.9)	
Richest	25,500	16.6 (15.9–17.4)		5240	28.4 (26.5–30.4)		4093	13.4 (11.9–15.1)	
Mass Media									
No	35,196	27.5 (26.8–28.3)	0.117/0.053 (0.817)	8992	28.9 (27.4–30.4)	45.390/17.585 (<0.001)	4786	24.1 (22.0–26.4)	14.110/5.851 (0.016)
Yes	145,549	27.5 (27.1–27.8)		24,670	32.7 (31.7–33.8)		21,037	21.2 (20.1–22.3)	
Toilet facility									
Improved toilet facility	131,067	27.0 (26.6–27.4)	47.094/20.420 (<0.001)	27,241	32.3 (31.3–33.3)	32.065/14.818 (<0.001)	22,741	21.0 (19.9–22.1)	34.514/10.219 (0.001)
Unimproved toilet facility	41,997	28.7 (28.1–29.4)		5466	28.2 (26.3–30.1)		2640	25.5 (22.8–28.3)	
Region									
North	34,623	23.7 (23.0–24.6)	402.45 (<0.001)	8965	31.6 (29.8–33.5)	84.49 (<0.001)	4960	21.4 (19.7–23.3)	48.15 (<0.001)
Central	56,102	37.7 (37.0–38.4)		7541	43.4 (41.6–45.2)		479	24.6 (21.3–28.3)	
East	34,777	27.4 (26.7–28.2)		6189	29.2 (27.4–31.1)		1863	33.8 (30.7–37.1)	
Northeast	11,448	37.0 (35.4–38.7)		4689	35.9 (33.6–38.4)		15,727	30.9 (29.2–32.7)	
West	18,067	15.4 (14.6–16.3)		2375	15.5 (13.5–17.6)		875	8.4 (6.5–10.8)	
South	25,728	20.2 (19.3–21.1)		3903	25.4 (23.3–27.6)		1919	17.9 (15.6–20.4)	

Note: Percentages are survey-weighted, whereas total numbers are unweighted sample sizes (n). Bivariate associations were assessed using the second-order Rao–Scott adjusted F test, accounting for the complex NFHS-V survey design, including sampling weights, strata, and primary sampling units (PSUs). Statistical significance was considered at *p* < 0.05. Totals may vary across variables because of missing observations. Caste/social-group and toilet-facility categories are based on respondents with available information for the respective variables; therefore, their category totals may not equal the total sample size for each religious group.

**Table 3:** Adjusted odds ratios (AOR) with 95% confidence intervals from a multivariable logistic regression examining the association between socio-demographic characteristics, religious affiliation, and reported dual menstrual material use, controlling for age, place of residence, marital status, education, caste/social group, household wealth, mass-media exposure, toilet-facility type, and region.

Predictor	Category	AOR	95% CI	*p* Value
Religion	Hindu (Ref.)	1	—	—
	Muslim	1.36	1.29–1.43	<0.001
	Others	0.91	0.85–0.98	0.015
Age	15–19 (Ref.)	1	—	—
	20–24	1.13	1.10–1.17	<0.001
Residence	Urban (Ref.)	1	—	—
	Rural	1.29	1.23–1.36	<0.001
Marital status	Never married (Ref.)	1	—	—
	Ever married	0.94	0.90–0.97	<0.001
Education	No education (Ref.)	1	—	—
	Primary	1.13	1.05–1.21	0.001
	Secondary	1.30	1.23–1.38	<0.001
	Higher	1.12	1.04–1.20	0.001
Social group	SC/ST (Ref.)	1	—	—
	OBC	1.04	1.00–1.08	0.032
	None of them	0.90	0.86–0.94	<0.001
Wealth index	Poorest (Ref.)	1	—	—
	Poorer	1.24	1.18–1.29	<0.001
	Middle	1.13	1.08–1.19	<0.001
	Richer	0.98	0.92–1.03	0.413
	Richest	0.67	0.62–0.72	<0.001
Mass media exposure	No (Ref.)	1	—	—
	Yes	1.29	1.24–1.34	<0.001
Toilet facility	Improved (Ref.)	1	—	—
	Unimproved	0.94	0.90–0.98	0.001
Region	North (Ref.)	1	—	—
	Central	1.78	1.69–1.87	<0.001
	East	1.09	1.03–1.16	0.003
	Northeast	1.52	1.41–1.64	<0.001
	West	0.56	0.51–0.60	<0.001
	South	0.74	0.69–0.79	<0.001

Note: Adjusted odds ratio (AOR) is the odds ratio adjusted for the effects of other factors in the model; CI, confidence interval; Ref., reference category. Estimates were derived using survey-weighted logistic regression with sampling weights, stratification, and clustering. Religion, age, residence, marital status, education, social group, household wealth, mass-media exposure, toilet facility, and region were included in the model. Analytic sample: 218,843 women. Population size: 218,345.85; 2710 strata; 29,612 PSUs; design degrees of freedom = 26,902.

**Table 4:** Adjusted predicted probabilities of reported dual menstrual material use by religious affiliation and selected characteristics.

Characteristic	Category	Hindu, % (95% CI)	Muslim, % (95% CI)	Others, % (95% CI)	*p* Value for Interaction
Residence	Urban	23.2 (22.4–24.0)	32.3 (30.5–34.1)	18.6 (16.3–20.9)	<0.001
	Rural	28.7 (28.3–29.1)	32.9 (31.7–34.1)	28.1 (26.6–29.7)	
Education	No education	23.5 (22.5–24.5)	28.6 (27.2–29.9)	22.0 (20.5–23.5)	0.031
	Primary	25.6 (24.6–26.6)	30.9 (29.5–32.3)	24.0 (22.5–25.6)	
	Secondary	28.2 (27.8–28.6)	33.8 (32.8–34.9)	26.5 (25.2–27.9)	
	Higher	25.5 (24.8–26.2)	30.8 (29.6–32.1)	23.9 (22.5–25.3)	
Wealth index	Poorest	26.9 (26.1–27.7)	32.5 (31.3–33.8)	25.2 (23.7–26.7)	<0.001
	Poorer	31.2 (30.5–31.8)	37.3 (36.0–38.5)	29.2 (27.7–30.8)	
	Middle	29.4 (28.7–30.0)	35.3 (34.1–36.5)	27.5 (26.0–29.0)	
	Richer	26.4 (25.7–27.2)	32.0 (30.8–33.2)	24.7 (23.3–26.2)	
	Richest	20.1 (19.3–20.9)	24.8 (23.5–26.0)	18.7 (17.5–20.0)	

Note: Predicted probabilities were estimated from the survey-weighted logistic regression model including the respective religion-by-characteristic interaction term, and adjusted for the other covariates in the model. Estimates account for the complex survey design of NFHS-V. *p* values represent design-adjusted tests of interaction between religious affiliation and the respective characteristic.

## Data Availability

The datasets analysed during the current study are available in the Demographic and Health Survey repository (https://dhsprogram.com/data/) (accessed on 2 February 2023).

## Abbreviations

MHM—Menstrual Hygiene Management; MHH—Menstrual Health and Hygiene; NFHS-V—National Family Health Survey, Round V (2019–21); IIPS—International Institute for Population Sciences; AOR—Adjusted Odds Ratio; CI—Confidence Interval; PSU—Primary Sampling Unit; SEM—Social Ecological Model; TPB—Theory of Planned Behavior; SC—Scheduled Caste; ST—Scheduled Tribe; OBC—Other Backward Classes; RTI—Reproductive Tract Infection; WHO—World Health Organization; UNICEF—United Nations Children’s Fund; UNESCO—United Nations Educational, Scientific and Cultural Organization; JMP—Joint Monitoring Programme (WHO/UNICEF); LMIC—Low- and Middle-Income Country.
